# Feasibility study and process evaluation of MRI plus physiotherapy vs. physiotherapy alone in non-specific chronic low back pain among patients in Saudi Arabia

**DOI:** 10.1186/s40814-020-00731-w

**Published:** 2020-11-30

**Authors:** Ahmed Alhowimel, Mazyad Alotiabi, Neil Coulson, Kathryn Radford

**Affiliations:** 1 Physical Therapy and Rehabilitation Science Department, Prince Sattam Bin AbdulAziz University, Alkarj, Saudi Arabia; 2 Division of Rehabilitation and Ageing, School of Medicine, University of Nottingham, Nottingham, UK

## Abstract

**Aim:**

To determine the feasibility of conducting a definitive randomised control trial (RCT) to answer the following questions: (1) Is early physiotherapy treatment acceptable and feasible for patients and direct healthcare providers? and (2) Is early physiotherapy intervention associated with better disability and psychosocial outcomes compared with the practice of routine MRIs?

**Methods:**

In a feasibility RCT in Riyadh City from 01 March 2018 until 29 July 2018, chronic low back pain (CLBP) patients presenting to spine clinics were randomised to receive an MRI (intervention) plus physiotherapy rehabilitation or physiotherapy alone (control group). The acceptability of randomisation to the control group (non-MRI) was tested during the recruitment by qualitatively interviewing study participants and referring physicians. Moreover, interviews with study participants explored the broader social, political, economic, and environmental (context) aspects that may influence trial delivery and intervention implementation.

**Results:**

The recruitment target was not met: 16/24 (66%) participants were recruited in 4 months (12.4% of those screened); 33% declined. The process evaluation identified numerous factors that may affect the success of a definitive RCT in Saudi Arabia. These were research resources, the lack of research infrastructure to support recruitment to trials, limited research capacity in terms of knowledge and skills of the healthcare team, and limited funding.

**Conclusion:**

A definitive RCT to test the influence of MRI diagnosis on the psychosocial and disability outcomes in people with CLBP treated with physiotherapy in Saudi Arabia is feasible. However, the lack of research infrastructure, research capacity, the impact of MRI on patient outcomes, and a lack of clinical equipoise in the treatment and management of CLBP in Saudi Arabia pose major barriers to clinical trials.

## Key messages regarding feasibility

- What uncertainties existed regarding the feasibility?

Compared to the routine use of MRI in spine clinics in Saudi Arabia, early physiotherapy treatment’s feasibility and acceptability are unknown.

- What are the key feasibility findings?

Sixteen out of twenty-four (66%) participants were recruited (12.4% of those screened); 33% declined, suggesting feasible randomisation. However, the process evaluation highlights other factors that might impede progressing to full RCT.

- What are the implications of the feasibility findings for the design of the main study?

Several factors may affect the success of a definitive RCT in Saudi Arabia. These are research resources, the lack of research infrastructure to support recruitment to trials, limited research capacity in terms of knowledge and skills of the healthcare team, and limited funding.

## Introduction

Although consensus in international guidelines is against the use of imaging to routinely diagnose low back pain (LBP) [1–3], several studies have identified an increase in the practice of imaging referral for diagnosis [4, 5]. This issue clearly indicates poor adherence among clinical practitioners to the established guidelines. Apart from the direct cost of imaging, the use of MRI for the diagnosis of LBP in patients has received considerable criticism. Not only does it lack the ability to identify the primary pathology [6, 7], but it is also prone to false-positive findings, resulting in a larger in the number of patient referrals to specialist facilities [8].

Several studies have investigated strategies intended to reduce the use of diagnostic imaging in the LBP population. Jenkins et al. [5] reported that modifying the referral form for MRI resulted in a 36.8% decrease in the rate of referrals. This suggests that referral for imaging is permitted for only three conditions, which are the presence of neurological abnormalities (hypoesthesia, hyperesthesia, or anaesthesia of lumbar or sacral dermatomes), weakness of the lower extremities (hyporeflexia), or bladder/bowel incontinence [5]. The other strategy, which achieved a 22.5% reduction in MRI use, was a targeted reminder to primary care general practitioners [5].

Kindrachuk and Fourney [9] suggested a promising programme to reduce the imaging rates in spine clinics, wherein a trained physiotherapist performed triage on new patients and only referred those with clear indications (for example, chronic steroid use or presence of neurological deficits) to the spine surgeon for surgery. For the others who had no indication of the need for spinal surgery, adequate education and self-management advice was provided. In addition, patients were triaged based on the STarT Back tool as the most appropriate guideline for recommended treatment. The preliminary data suggested a reduction in referrals to imaging and waiting time to see a surgeon [9].

Providing an alternative treatment option for patients has been suggested as a strategy to address the idea of acceptability [10]. Physiotherapy services are known to be a credible and acceptable option for many patients [11–13]. When comparing LBP patients who underwent MRI at the first visit to a primary care clinic in the USA with those who received a physiotherapy intervention, Fritz et al. [11] showed that the cost of care was on average $4,793 higher over 1 year for the immediate MRI group than the physiotherapy group. Numerous studies have suggested that rapid physiotherapy intervention for LBP is linked to better patient outcomes and greater satisfaction [14, 15]. Moreover, the direct costs associated with LBP—including the number of MRI scans, GP visits, and prescription medications—were lower for people with chronic LBP (CLBP) who received early physiotherapy intervention than for those who did not [14–17].

This evidence suggests that early access to physiotherapy treatments may prevent unnecessary medical expenditure while also improving the degree of patient satisfaction. On the other hand, delayed access to physiotherapy for CLBP patients is linked to poor outcomes, as seen by higher disability scores, a delayed return to work, less satisfaction, and increased chronicity [18–20].

The standard practice in spine clinics involves the routine use of MRI in CLBP diagnosis, despite its minimal effectiveness in clinical decision making [21]. It is not known whether altering this practice of routine MRI scanning and offering early access to physiotherapy would be acceptable to healthcare practitioners and patients and lead to improved psychosocial and disability outcomes. Therefore, this study aims to answer the following questions: (1) Is early physiotherapy treatment acceptable and feasible for patients and direct healthcare providers? and (2) Is early physiotherapy intervention associated with better disability and psychosocial outcomes compared with the practice of routine MRIs? The primary objective of this study is related to the feasibility of an RCT, specifically (1) willingness for participation, (2) recruitment rate and eligibility, and (3) acceptability of randomisation. The secondary objective is to examine if altering the practice is associated with better disability and psychosocial outcomes, evaluated using patient-reported outcome measures.

This study will seek to examine the feasibility and acceptability of conducting an RCT to fulfil the following progression criteria:

1. Recruitment: recruit at least 24 participants in the two arms
2. Follow-up: if there is no more than 20% loss to follow-up
3. Acceptability: if most participants interviewed stated that randomisation is acceptable and if at least 65% of eligible patients consent to participate in the trial

## Method

### Ethical approval and registration

This study conformed to the Consort 2010 statement [22] of reporting feasibility and pilot studies. Ethical approval was obtained from the Research Ethics Committee of the Faculty of Medicine and Health Science at the University of Nottingham (Ethics Reference Number: OVS 18082016) and King Fahad Medical City in Riyadh, Saudi Arabia (IRB: H-01-R-R-012).

The study is registered in the ISRCTN registry with the registration number ISRCTN14405580.

### Method

This study was designed as a single-centre, two-arm feasibility RCT in Riyadh City from 01 March 2018 until 29 July 2018 and used the opaque envelope technique. Following a baseline assessment, participants were randomly allocated to one of two arms. In the intervention arm (MRI + physiotherapy), the participant was referred for an MRI and the results were discussed with the referring doctor, followed by a referral to physiotherapy if no serious pathology was detected by the MRI. In the control arm (physiotherapy alone), all participants were referred directly to physiotherapy without having an MRI scan.

### Participants

Participants were eligible for inclusion if they satisfied the following criteria: participants of either sex aged 18–65 years, complaining of CLBP with no apparent medical diagnosis, and pain persisting for more than 3 months.

The following exclusion criteria were applied: pregnant women, new mothers (< 6 months postpartum), those who had undergone pain-relieving procedures (injection or denervation) in the previous 3 months, those who showed evidence of neurological impairment specific to LBP, patients with an established clear medical diagnosis (malignancy, fracture, infection, spinal stenosis, spondylolisthesis, or inflammatory disease), and patients who had received physiotherapy treatment for their LBP and/or an MRI scan in the 6 months before recruitment.

### Recruitment

The initial plan was to begin recruitment from a governmental and private hospital. However, the ethics committee of the large private hospital declined participation because the research was contrary to their practice of routinely scanning CLPB patients. Therefore, the study was conducted in one only centre—King Fahad Medical City, Riyadh. The centre is a tertiary-care hospital in Saudi Arabia and receives referrals from secondary and primary care centres across the country. A member of the usual care team in the spine clinic screened all new patients for inclusion in the study. All patients who satisfied one or more of the exclusion criteria were excluded, and the reason for their exclusion was recorded.

Potential participants who fulfilled the inclusion criteria were recruited only after informed consent had been obtained by the principal investigator (AA) during their initial visit to the spine clinic. Several measures were implemented to achieve this: first, contact was established with prospective patients’ healthcare providers (members of the usual care team) to provide information about the study and to request that they provide potential participants with an explanation of the study’s aims and purpose, a description of what would be involved, and hand out participant information sheets to those fitting the inclusion criteria. Second, those fitting the inclusion criteria and expressing an interest in participating were approached by AA and given the opportunity to ask any questions. Once any questions had been answered satisfactorily, written informed consent was obtained by AA from all patients, and baseline measurements were performed.

Concealing the approach to group allocation adopted in this research from either the participants or the health professionals was not possible.

### Blinding

A successful approach to blinding would have required an overly creative and resource-intensive strategy that was not practical for this study. In view of these considerations, blinding was not applied in the present study.

### Randomisation

Simple randomisation took place using opaque sealed envelopes containing group allocation. To ensure concealment of allocation, an independent non-healthcare practitioner, who was not part of the research team and had no access to participants’ information, sealed and numbered each envelope.

### Intervention

Participants allocated to the intervention arm were sent for an MRI of the lumbar spine, and a follow-up visit was planned to discuss the results. The time interval to undergo the MRI ranged from 3 to 6 weeks. After discussing the results with their doctor, participants were referred to physiotherapy, which lasted for a period of 2 to 4 weeks. The MRI scan has no value in planning physiotherapy treatment for patients with CLBP, yet it is used routinely despite clinical guideline recommendations. As a pragmatic trial, physiotherapy was not predetermined for both arms but rather followed whatever interventions were routinely delivered in Saudi Arabian clinical practice. This could include passive treatment, such as mobilisation and electrotherapy, or active treatment, such as exercises and advice to stay active.

Following allocation, participants in the control arm were immediately asked to complete the booklet of questions and standard questionnaires. They were then referred to a physiotherapist for treatment, and the time required to initiate physiotherapy ranged from 1 to 2 weeks. After completing the physiotherapy treatment programme, which lasted for 2 to 4 weeks, the second assessment was carried out.

Baseline data were collected by AA or the nurse in charge, and all endpoint data were collected in both arms by AA on completion of the physiotherapy treatment.

Demographic data (collected at baseline) included age, gender, marital status, number of children, employment status, monthly income, highest educational level, duration of back pain, number of sick days in the last 3 months, the severity of back pain in the last 3 months, and any history of surgery.

### Sample size

The sample size (*n* = 36) was calculated from the number of patients matching the inclusion criteria who visited spine clinics in the 3 months before conducting the study. The estimated non-consent rate of 50% suggested six new patients to be randomised per month, giving 24 patients over the 4 months proposed recruitment time.

### Outcome measures

Patient-reported outcome measures were used as a secondary objective of this study to examine if altering the practice would result in better outcomes. Four standardised outcome measures were completed at the baseline and after physiotherapy intervention.

#### Roland Morris Disability Questionnaire (RMDQ)

This is a self-administered tool for assessing the level of physical disability caused by LBP. The reliability and validity of the RMDQ have been reported to be effective with an intraclass correlation coefficient (ICC) of 0.089 [23]. It demonstrated good construct validity and good correlation with the numeric pain scale (*r* = 0.71) [23]. The cross-cultural adaptation and translation of the RMDQ to the Arabic language is also reported to have good reliability: ICC = 0.092, with high internal consistency Cronbach *α* of 0.729 [24].

#### Fear-Avoidance Beliefs Questionnaire (FABQ)

This questionnaire is based on the fear-avoidance model [25]. Its purpose is to measure the degree to which patients are fearful of physical activities and, thereby, avoid them, along with the impact of this avoidance on their activities. The tool consists of 16 items scored from 0 (low fear) to 6 (high fear).

#### Örebro Musculoskeletal Pain Questionnaire (ÖMPQ)

This questionnaire was developed to guide the primary care clinician in the identification of patients at high risk of persistent back pain [26]. It is widely used and recognised in clinical guidelines for back pain management [26]. The questionnaire has been translated into Arabic in a previous study on the Saudi Arabian population [27], but the validity of the translated version is not reported in the literature.

#### Back Beliefs Questionnaire (BBQ)

This questionnaire was developed to assess patients’ beliefs relating to back pain and recovery. It is notable that the questionnaire has been translated and cross-culturally adapted to the Arabic population [28].

### Data analysis

Given that this was a feasibility study, data analysis was primarily concerned with reporting feasibility outcomes using descriptive statistics. This included sociodemographic information pertaining to the participants, the number of dropouts, the number of participants who refused consent, and the rate of loss associated with follow-up. Continuous variables are summarised as mean (SD) if normally distributed or median (range) for skewed data. Categorical data are summarised as counts and percentages.

### Process evaluation

The process evaluation utilised both quantitative data from the recruitment process collected by the research team and qualitative data from participants’ interviews.

In an ethnographical qualitative research approach, semi-structured interviews were used to explore the acceptability of the study protocol to both patient participants and healthcare practitioners involved in the trial delivery (Appendix). Since the process evaluation was set to explore the results of the feasibility outcomes, we invited seven participants (five patients and two doctors), representative for age and gender, for a one-to-one interview. All interviews were audio-recorded and transcribed verbatim by AA. Furthermore, a thematic analysis approach was used [29] using NVivo software. The validity of the obtained data was assured by data triangulation, whereby a summary of the findings was communicated with 20% of the qualitative interview participants.

The participating centre was described in terms of the number of doctors, supporting staff, and the caseload. The number of participants was recorded to highlight the rate of participation, attrition, and dropout.

## Results

**Fig. 1:**
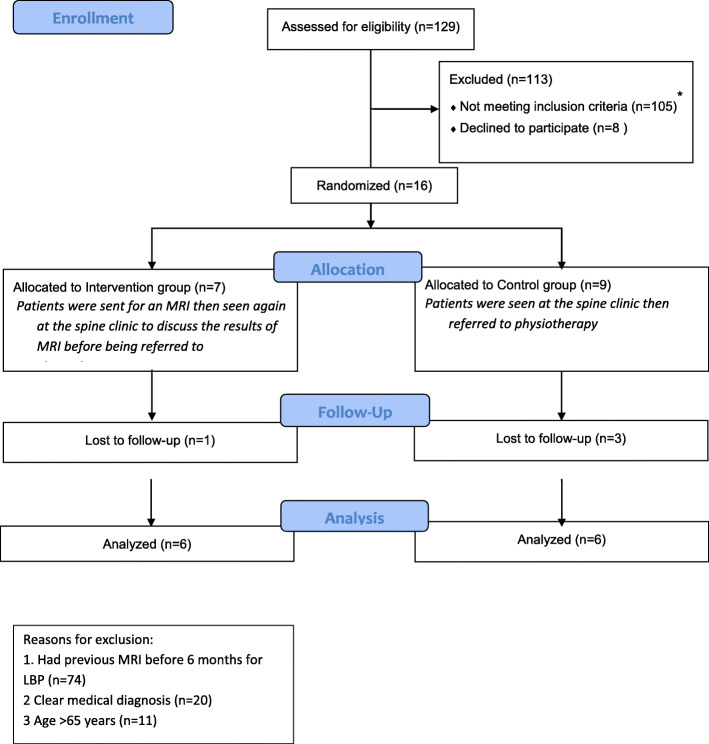
*CONSORT flow diagram*

In all, 129 patients were screened over the 4-month recruitment period. Of these, 24 (18.6%) satisfied the inclusion criteria (Fig. 1). Eight (33.3%) of these 24 did not wish to participate in the study. Some did not state their reason (*n* = 5), others attributed their decision to the time-consuming nature of completing the outcome measure booklet (*n* = 2), and the remaining patient (*n* = 1) was dissatisfied with the concept of randomisation. Of those consenting to take part in the study, five (41.6%) further consented to participate in the process evaluation interview.

The demographic characteristics of the included patients are shown in Table 1.

**Table 1:** Participants’ characteristics

	Control group, *n* = 7 (43.75%)	Intervention group, *n* = 9 (56.25%)
Age, mean (S.D, range)	40.57 (10.77, 22–52)	37.78 (11.25, 24–55)
History of back pain (months) mean (S.D, range)	54.86 (27.59, 24–96)	32.67 (17.78, 6–72)
Initial pain level (out of 10), mean (S.D, range)	6.00 (1.15, 1–7)	5.88 (1.83, 3–9)
Gender (number, %)		
Female	6 (85.7)	5 (55.6)
Level of education (number, %)		
Primary school	1 (14.3)	2 (22.2)
Secondary school	1 (14.3)	1 (11.1)
College/diploma	2 (28.6)	1 (11.1)
University degree	2 (28.6)	4 (44.4)
Postgraduate	1 (14.3)	1 (11.1)
Occupational status (number, %)		
Employed full-time	1 (14.3)	6 (66.7)
Employed part-time	1 (14.3)	1 (11.1)
Retired	1 (14.3)	0 (0.00)
Unemployed	2 (28.6)	0 (0.00)
Housewife	2 (28.6)	2 (22.2)

Out of the 105 excluded patients, 74 (57.3%) underwent MRI examination within the preceding 6 months, and 20 (15%) had a specific medical diagnosis (fracture, cancer, or lumbar stenosis) that excluded participation in the study. Of those screened, 11 (8.3%) were over the age of 65 years, which was one of the exclusion criteria.

### Treatment outcomes

Regarding baseline measurements, both groups showed similar levels of disability based on RMDQ scores (between 8.57 and 8.88). However, FABQ-W was markedly higher in the control group (19.23; SD 9.14 compared to 14.71; SD 4.64) (Table 2).

**Table 2:** Outcome measure scores for both groups

Outcome measures	Intervention group (PT alone)		Control group (MRI + PT)	
	Baseline	After PT	Baseline	After PT
RMDQ (mean, S.D)	8.57 (4.43)	9.00 (4.29)	8.88 (5.41)	9.16 (3.86)
FABQ-W (mean, S.D)	14.71 (4.64)	9.28 (4.71)	19.24 (9.14)	15.55 (13.65)
FABQ-P (mean, S.D)	18.42 (12.32)	12.42 (9.3)	16.22 (4.81)	10 (8.71)
QMPQ (mean, S.D)	62.00 (10.59)	69.50 (10.40)	57.66 (11.63)	59.66 (12.50)
BPB (mean, S.D)	26.57 (5.62)	27.16 (6.73)	29.66 (4.61)	26.33 (5.50)

*PT physiotherapy, RMDQ* Roland Morris Disability Questionnaire, *FABQ-P* Fear-Avoidance Beliefs Questionnaire Personal*, FABQ-W* Fear-Avoidance Beliefs Questionnaire Work, *BPB* Back Beliefs Questionnaire, *OMPQ* Orebro Musculoskeletal Pain Questionnaire

In a follow-up measure, both RMDQ and QMPQ displayed a marginal increase after intervention in both groups, ranging from 0.43 to 0.28 in RMDQ and 6.5 to 2 in QMPQ. Moreover, all groups showed a decrease in FABQ-P and FABQ-W following the intervention, ranging from 6.24 to 4.43 points.

### Process evaluation

A total of seven participants (five patients and two spine surgeons) agreed to participate in the process evaluation one-to-one interviews. Three main themes were identified from the interviews.

#### Acceptability

The responses from trial participants enrolled in this study suggest that a diagnosis based on imaging and seeing a doctor was still important to patients with LBP.

I do not think they will accept it, according to my experience. I am a patient with lower back pain. Anyone who suffers from lower back pain wants first to be treated by a doctor, which means he needs imaging to see what is going on inside. PT5

However, some patients were not concerned about the group to which they were allocated, on the grounds that they had already undergone several MRI scans.

Being in either group was not a big issue for me as I have had many MRIs before. PT3

#### Satisfaction

At the same time, it is important to recognise that the implementation of a large-scale study may have an adverse impact on the degree to which patients are satisfied with the healthcare they receive. One doctor respondent in this study drew attention to this issue when the matter of feasibility was presented to him.

Any patient wants to be in the group that is getting the most tests, diagnostics, and treatment. I don’t think patients will be satisfied. Dr. 1

Furthermore, it was suggested that LBP patients’ referrals to the spine clinic might be reduced if they received physiotherapy as part of their primary care.

Seventy percent of the cases that we see here could be treated by family doctors and physiotherapy. Dr. 2

#### Contextual factors

The absence of additional help for recruitment and to support baseline data collection eliminated the option of conducting a multicentre study, since additional human resources would have been required for the purpose of the screening, identification, and recruitment of participants and completing the baseline measures and randomisation.

The only obstacle I foresee is the lack of researchers or assistant researchers; hospitals are very supportive if you want to conduct research. Dr.2

Time limitations are always problematic when attempting to collect clinical data, especially for doctors. This is because they rarely receive exemptions from normal working duties for research purposes, and they have to do this in their free time.

If you are looking for a large study with follow-up, then we cannot give our own time to research. Dr.1

## Discussion

This study aimed to evaluate the acceptability and feasibility of allocating non-specific CLBP patients randomly to an intervention (MRI) or control (non-MRI) group in addition to physiotherapy treatment. The main finding of this study is that randomisation was possible; however, multiple factors emerged from the qualitative interview that might hinder proceeding to a definitive RCT. Although 16 patients were recruited to the study in 4 months, eight more patients were required to reach the required total of 24. Hence, the study fell short of achieving the recruitment target by 33%, which may be attributed to the study setting being limited to one governmental hospital. Moreover, the location of the study could be considered to be one of the barriers to participation, as indicated by Mills et al. [30]: patients in a rural area might find it difficult to participate in RCT studies due to transportation challenges.

It should be noted that the median recruitment rate of RCTs in the UK was found to be 0.92 participants per month per centre [31]. An insufficient recruitment rate has been reported in multiple RCTs on LBP [32–34]. This finding is very valuable in highlighting the fact that researchers should not be overly optimistic with respect to recruitment rates when designing definitive RCTs. Furthermore, we found no RCT conducted in Saudi Arabia that reported the recruitment rate.

Despite the inability to recruit the proposed number of patients (*n* = 24), this study was successful in randomly allocating people to undergo MRI in addition to physiotherapy treatment, suggesting that recruitment materials (i.e. the patient information sheet) were effective in explaining the study and that the idea of random allocation was acceptable.

Moreover, it is notable that while the minimum permissible range of non-consent rate is not subject to standardisation, 40% may be considered unacceptable [35]. The present study’s non-consent rate of 33% was considered acceptable when compared to that reported in the available LBP literature [36].

The risk of bias is regarded as considerable when the loss to follow-up exceeds 20% [37]. The loss to follow-up rate in the present study was 25% (*n* = 4). However, this was dependent on the availability of participants attending their final session of physiotherapy. Therefore, it is not known whether this is truly reflective of retention at the follow-up point or adherence to the physiotherapy intervention. The limited duration of this study (4 months), owing to the chief investigator’s (AA’s) visa restrictions, meant that it was not possible to test the additional effectiveness of other follow-up methods, such as online or telephone data collection, or text message prompts to boost follow-up. The lack of funding for the trial and lack of research infrastructure, including trained research nurses to assist with recruitment and data collection, meant that all follow-up depended on the efforts of AA. Follow-up assessments would likely be better in a properly resourced trial.

The delay in the start of physiotherapy intervention in participants in the MRI group (compared to their counterparts in the non-MRI group) could introduce potential bias into the study findings. However, delayed physiotherapy treatment is one consequence of routine scanning for patients with LBP.

Screening prior to recruitment involved the examination of patient files in the recruitment centre by one of the usual care teams. It is possible that patients had had an MRI at another healthcare centre, leading to contamination in the non-MRI group. This was highlighted by one of the control participants in the process evaluation interview, who stated that he was not concerned about being allocated to the non-MRI group having undergone numerous previous MRI exams. In a future trial, the mechanism for cross-referencing with electronic data records and confirmation with potential participants themselves should form part of the screening and consent procedures.

It might seem that doctors are supportive of recommending physiotherapy alone as a primary treatment for LBP; however, they are only concerned with patients’ satisfaction. It should be noted that the inclusion of patient satisfaction measures is encouraged if full RCT is to be implemented.

The secondary aim of this research was to estimate appropriate values for a power calculation for a definitive trial. The present study’s small sample size, along with the high dropout rate, did not warrant a power calculation [38]. However, it is plausible to draw on comparable studies that had a larger sample size [35]. The RMDQ is one of the primary outcome measures used in this study and has been used in previous research to calculate the mean baseline and minimal clinically important change required for calculating the sample size [39–41]. Using this measure, a sample size of 136 in each arm would have 90% power to detect a difference in mean of 2.5 points in the RMDQ scores between the intervention and control groups. Assuming a mean of 9.7 and SD of 5.6 points in the control group, with a two-sided significance level of 0.05 and a sample size inflated by 25% to account for loss of follow-up, would require 340 participants with CLBP. Based on this feasibility study and allowing 12 months for recruitment, we would require at least seven centres with spine clinics to screen approximately 2709 people with CLBP for eligibility.

The findings of this feasibility and process evaluation study provide valuable insights for researchers planning to conduct a clinical trial for CLBP treatment in Saudi Arabia.

Various barriers limit the feasibility of conducting a definitive RCT to test the influence of MRI diagnosis on psychosocial and disability outcomes in people with CLBP treated with physiotherapy in Saudi Arabia. A large trial would require multiple recruitment centres, a lengthy recruitment period, and research staff trained in good clinical practice. Moreover, physician and participant concerns surrounding the acceptability of randomising patients not to receive an MRI may limit success and suggest that progressing to a large-scale RCT would be impractical at the present time.

## Conclusion

Although the data suggests feasible recruitment and randomisation for a definitive trial, it would invariably face the following obstacles: time limitations, the need to find a greater number of recruitment centres, the requirement to employ a research assistant, and, in view of the previous point, substantial funding. Additionally, randomisation might be less acceptable as an MRI scan is imperative to diagnose CLBP in Saudi Arabia.

## Supplementary Information

**Additional file 1.** Appendix

## Data Availability

The datasets used and/or analysed during the current study are available from the corresponding author on reasonable request.
