# Supplementary material for: Feasibility study and process evaluation of MRI plus physiotherapy vs. physiotherapy alone in non-specific chronic low back pain among patients in Saudi Arabia
Source: Pilot Feasibility Stud. 2020 Nov 30;6:188. doi: 10.1186/s40814-020-00731-w (PMC7702682; doi:10.1186/s40814-020-00731-w)
Supplement: Supplementary file 1 — Additional file 1. Appendix [file 40814_2020_731_MOESM1_ESM.docx]

Appendix 1

**Framework for interviews with clinician**

- Thinking about the use of MRI for NS-LBP, do you feel that this tool is essential in the management of such cases? Please explain your answer.
- What factors do you think can influence the decisions of whether to order MRI for NS-CLBP?
- Is it acceptable to randomise patients into MRI groups and non-MRI groups? Please explain your answer.
- Is it feasible to randomise patients into MRI groups and non-MRI groups that work with other agencies, such as the Ministry of Health, within the private sector or at military hospitals? Please explain your answer.
- Think about larger context of the study: in your opinion, what are the social or political issues that could impact the study’s design?
- Can you identify any social or political barriers that would *prevent* the feasibility study from being undertaken?
- Do you think your current clinical role encourages involvement in research practices?
- Considering the research environment, did you find you were supported when conducting the research? If so, what kind of support did you receive? Please explain.
- Are there any other comments you wish to add?

**Framework for interviews with NS-CLBP Patients:**

***Questions will be asked on the following topics, some examples of which are given below.***

| **Construct** | **Question** | **Prompts** |
| --- | --- | --- |
| **NS-CLBP specifics** | Tell me something about your back pain and how things have been going for you. | - How does the LBP affect you? (physically, thinking skills, emotionally, socially) - How does the LBP affect your work situation? (relationships, capacity to work) |
| **Acceptability** | What made you participate in this randomised study? | - Have you had any concerns about randomisation? - Do you think this method will be accepted by patients with LBP? |
| **Recruitment -** **utility of materials/measures and recruitment methods**  *As a participant in the study, you were asked to complete questionnaire booklets at the recruitment stage and again after physiotherapy.* | Did you encounter any problems with the information you were given to read? Please explain. | - Did you encounter any problems completing and returning the questionnaires? Please explain. - Was it written in an understandable language? - In terms of time to complete the questionnaire, how long did it take you to fill it in? - Were you given any help with its completion? - Was there any question/s that you felt were inappropriate? |
| **Context** | Do you think that there will be difficulties in conducting this study? | - Regarding the waiting time for results and a referral for physiotherapy, was the waiting time appropriate for you? Please explain. - Considering the communication between you and the research team, were you comfortable being contacted several times during the study? - Have you received any conflicting information from the research team regarding the study? |

**Additional Comments**

Is there anything else you would like to add or comment regarding your participation in the study?
